# Mouse models for microphthalmia, anophthalmia and cataracts

**DOI:** 10.1007/s00439-019-01995-w

**Published:** 2019-03-27

**Authors:** Jochen Graw

**Affiliations:** Institute of Developmental Genetics, Helmholtz Center Munich, German Research Center for Environmental Health, Ingolstädter Landstrasse 1, 85764 Neuherberg, Germany

## Abstract

Mouse mutants are a long-lasting, valuable tool to identify genes underlying eye diseases, because the absence of eyes, very small eyes and severely affected, cataractous eyes are easily to detect without major technical equipment. In mice, actually 145 genes or loci are known for anophthalmia, 269 for microphthalmia, and 180 for cataracts. Approximately, 25% of the loci are not yet characterized; however, some of the ancient lines are extinct and not available for future research. The phenotypes of the mutants represent a continuous spectrum either in anophthalmia and microphthalmia, or in microphthalmia and cataracts. On the other side, mouse models are still missing for some genes, which have been identified in human families to be causative for anophthalmia, microphthalmia, or cataracts. Finally, the mouse offers the possibility to genetically test the roles of modifiers and the role of SNPs; these aspects open new avenues for ophthalmogenetics in the mouse.

## Introduction

Blindness in children is a very severe condition affecting ~ 14 million children worldwide (Solebo et al. 2017). Among them, cataracts are the major subgroup affecting 28% of the cases (Solebo et al. 2017); the prevalence of congenital cataracts ranges from 0.32 to 22.9/10,000 children in different geographic regions over the world (Sheeladavi et al. 2016). Similarly, the prevalence of anophthalmia/microphthalmia has been estimated between 0.2 and 3.0 per 10,000 births (summarized by Llorente-González et al. 2011) with an incidence of congenital anophthalmia (in England) ranging from 0.4 to 2.9 per 100,000 infants and an incidence of congenital microphthalmia ranging from 10.0 to 10.8 per 100,000 children (Dharmasena et al. 2017). Although these eye disorders are rare, they represent a major challenge for the treating clinicians and for the families, who have to fight the diseases. Discussing the reasons for such severe disorders, we have to consider prenatal environmental factors such as intrauterine infections or toxins, but also genetic reasons. Here, we will focus on these genetic aspects leading to anophthalmia, microphthalmia or congenital cataracts. Summaries of the human cases have been published recently (e.g. Reis and Semina 2015; Anand et al. 2018); therefore, in this review, I will concentrate on mouse models, because the mouse is genetically the best characterized mammalian model system for hereditary diseases, particularly, if they affect the eye.

Anophthalmia, severe microphthalmia and congenital cataracts are indicators of major disturbances during early embryonic eye development. Briefly, in mice, eye development starts around embryonic day 9.5, when the lens placode thickens getting close to the underlying neural ectoderm of the diencephalon. The lens placode invaginates forming the future lens; thereby, it separates from the surface ectoderm, which will form the future cornea. At the same time, when the lens placode invaginates, also the underlying neural ectoderm of the diencephalon invaginates, and its inner part will form the various layers of the neural retina, whereas the outer part will give rise to the retinal pigment epithelium. When the mouse is born, lens and cornea are fully developed, but the eye lids are still closed, and the retina needs another 2 weeks to maturation. Two weeks after birth, the mice open their eyes and they are ready for life. More detailed reviews on the molecular mechanisms of eye development have been published recently (Anand and Lachke 2017; Cvekl et al. 2015; Reis and Semina 2015; Graw 2010).

Anophthalmia and severe microphthalmia are mainly caused by aberrations during early eye development; in human ophthalmogenetics, they are discussed frequently together with coloboma as “microphthalmia, anophthalmia, coloboma spectrum” (Reis and Semina 2015). However, colobomata are very specific defects of the formation and closure of the choroid fissure. Even if defects in the choroid fissure are responsible for up to 10% of pediatric blindness (Bernstein et al. 2018), it is rather difficult to find it in mice. Only a few mouse mutant lines are known like the *Pax2* mutants suffering from the renal coloboma syndrome, because *Pax2* is expressed not only in the developing eye, but also in the kidney leading eventually to defects in both organs, if mutated (Favor et al. 1996). Actually, 12 *Pax2* alleles are listed in the MGI database, and in 5 of them, the renal coloboma syndrome has been reported (http://www.informatics.jax.org/allele/summary?markerId=MGI:97486).

Anophthalmia, severe microphthalmia and strong cataracts are easily to detect in the mouse by investigators and, therefore, they are discussed together here. It is not surprising that some of the first mouse mutants with such severe eye defects were published already in the first half of the twentieth century (Little and Bagg 1923; Hertwig 1942). Later, several systematic screens were conducted using ionizing radiation or ethyl-nitroso urea (ENU) as mutagenic agents to identify disease-causing genes including genes leading to eye disorders (Kratochvilova 1981; Ehling et al. 1985; Hrabé de Angelis and Balling 1998; Acevedo-Arozena et al. 2008; Clark et al. 2004; Aigner et al. 2011). These programs have been very successful in the identification of novel genes; today, ENU screens to detect novel genes are still performed at the Riken Center (Japan; http://www.riken.jp/en) and by the Bench-to-Bassinet program (b2b; http://www.benchtobassinet.com). However, targeted and/or conditional mutagenesis is performed nowadays more frequently to better understand the underlying mechanisms during mammalian eye development. Another source for the identification of novel genes involved in early eye development is the mouse phenotyping centers, which systematically screen targeted knockout mutants for a broad variety of pathological phenotypes including the eye (http://www.mousephenotype.org/). The review here summarizes the genetic characterizations of mouse mutants suffering from microphthalmia, anophthalmia, and cataracts—models leading to a better understanding of eye development in mammals. New resources will be discussed to complete the mosaic.

## Anophthalmia

Anophthalmia is the most severe phenotype, which might occur during eye development, since it indicates that eye development stopped at a very early stage in one or both eyes. In humans, the genetic cause for anophthalmia (and severe microphthalmia) can be identified in ~ 80% of cases because of mutations in *SOX2* or *OTX2* (Williamson and FitzPatrick 2014). For the mouse, the mammalian phenotype ontology annotation lists 145 genes being associated with anophthalmia (just 28 mutations are unknown). Some of them, e.g. *Rax, Sox2, Pax6, Lhx2* and two *Bmp* genes (*Bmp4* and *Bmp7*) are well characterized in the mouse.

One of the early anophthalmic mouse mutants was identified by Herman Chase (1944) and referred to as eyeless (gene symbol *ey*). It took almost 60 years, till the mutation was characterized: it is a point mutation affecting the ***Rax*** gene (retina and anterior neural fold homeobox); it changes the Met at pos. 10 to Leu (M10L) (Tucker et al. 2001). The *Rax*^*ey*^ allele is hypomorphic, since the homozygous mutants are fully viable; in contrast, the homozygous knockout mutants of *Rax* are perinatally lethal (Mathers et al. 1997). The reason for this difference could be the type of the mutation leading to a conservative exchange of Met by Leu, which also affects an alternative, but in-frame translational start site. The authors argue that the *Rax*^*ey*^ allele leads to a reduced translation, but not to a real null allele (Tucker et al. 2001).

Another important gene for mammalian eye development is ***Sox2*** [SRY (sex-determining region Y)-box 2]. Mutations in human *SOX2* lead to anophthalmia, but most mutations are de novo mutations, which appear in the parental germline, and most of the affected persons are sterile (Fantes et al. 2003; Ragge et al. 2005; Bakrania et al. 2007). Therefore, it is not surprising that no spontaneous mouse mutant of this gene exists. On the other hand, several targeted mouse mutants are available: most of them act as hypomorphic alleles (*Sox2*^*LP*^, *Sox2*^*IR*^, *Sox2*^*EGFP*/IR^ and *Sox2*^*EGFP*/LP^) and show a range of eye phenotypes from mild microphthalmia to severe anophthalmia. The *Sox2*^*EGFP*^ mutant is a full knockout with the enhanced GFP replacing the coding sequence of the *Sox2* gene. The *Sox2*^*LP*^ allele contains a *loxP* site 5′ of the locus, and the *Sox2*^*IR*^ has IRES and dsRED2 coding sequenced instead of the 3′-UTR of the *Sox2* gene. Both alleles show < 40% activity as compound heterozygotes (*Sox2*^*EGFP*/IR^ and *Sox2*^*EGFP*/LP^). The severity of the phenotype depends on the remaining *Sox2* expression suggesting a dose-dependent function of Sox2 during early eye development (Taranova et al. 2006).

**Fig. 1:**
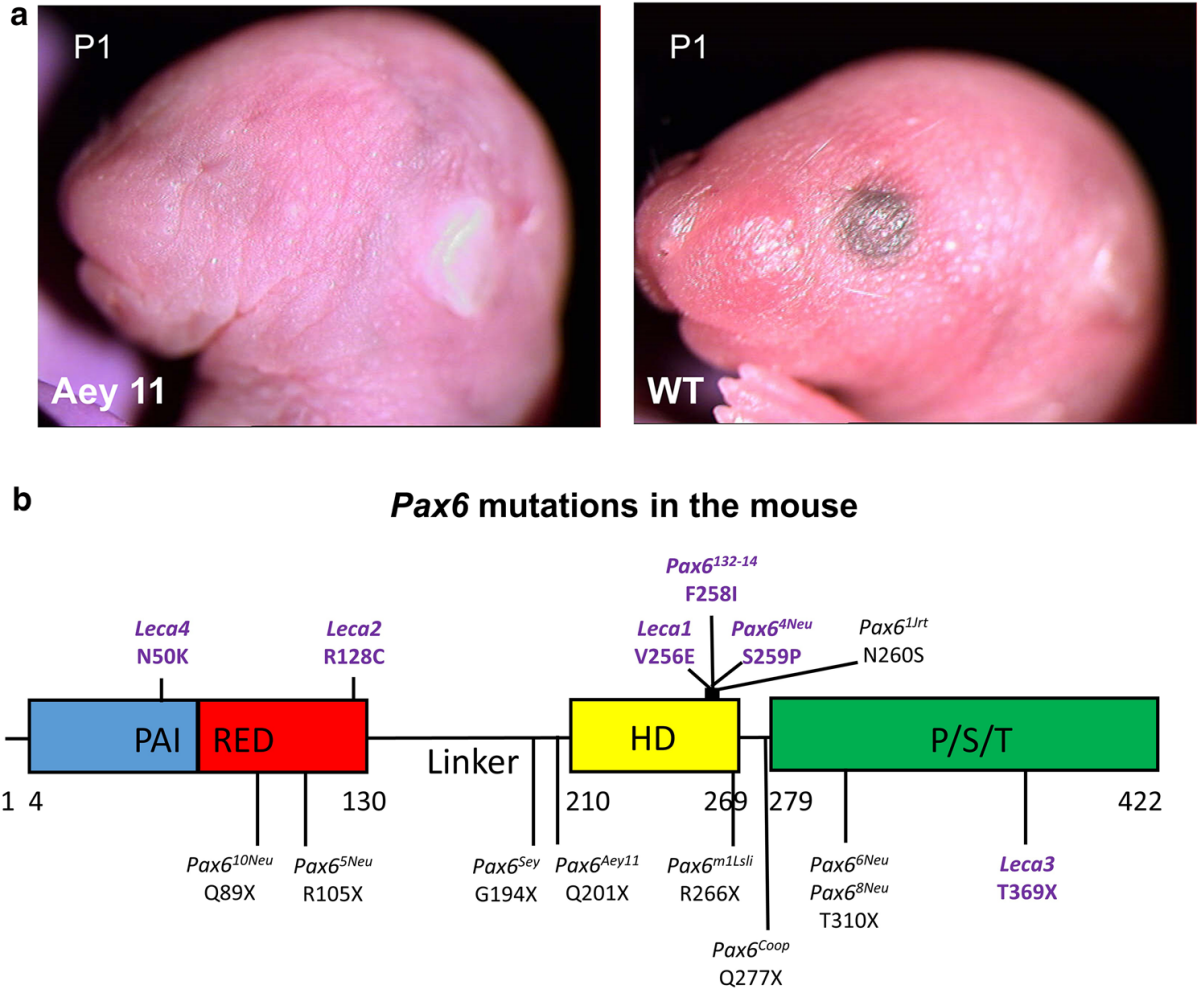
Anophthalmia mouse mutant. **a** Head of a neonatal (P1) homozygous *Pax6*^*Aey11*^ mutant compared to a wild-type mouse (wt) at the same age. The absence of eyes in the mutant is obvious. The eyelids of neonatal mice are still closed (photography: Jana Löster†, unpublished). **b** The major structural domains of the transcription factor PAX6 are the paired domain (with a N-terminal and C-terminal subdomain; here referred to as PAI—RED), a linker region to the homeodomain (HD), and finally a proline–serine–threonine-rich sequence (P/S/T); the amino acid position at the beginning and the end of the domains are given according to UniProtKB—P63015 (PAX6_MOUSE; feature viewer) for the canonical PAX6 protein of 422 amino acids (i.e. without exon 5a). Point mutations with amino acid exchanges are depicted above the scheme, and point mutations leading to premature stop codons are given below (not to scale). The allele names and the positions of the changed amino acids are given according to the MGI database; the amino acid positions in the database were re-calculated for the canonical protein without exon 5a, if necessary. Hypomorphic mutations are given in purple. Mutations leading to hybrid proteins (splice-site mutations, deletions, insertions) are not shown

The classical paradigm for anophthalmia in the mouse, however, is the homozygous ***Pax6*** mutants (paired box 6), which do not develop eyes in homozygous mutants (Fig. 1a). The first mouse mutant suffering from a *Pax6* mutation was detected as a dominant homozygous lethal mutation with a “small-eye” phenotype as heterozygotes (gene symbol *Sey*; Roberts 1967); the homozygous *Sey* mutants die perinatally. Another small-eye phenotype was found in 1975 at Harwell (UK) among offspring whose parents (fathers) have been treated by irradiation. Both mutations, *Sey* and *Sey*^*H*^ (for Harwell), were mapped to mouse chromosome 2 and shown to be allelic (Hogan et al. 1986). Finally, the *Sey*-mutation was identified as a G→T transversion in codon 194 of the *Pax6* gene changing the corresponding position in the protein from a Gly to a stop codon, which results in a premature termination before the homeobox domain. The *Sey*^*H*^ allele was characterized by a major deletion affecting not only *Pax6*, but also some adjacent genes (Hill et al. 1991). In the meantime, the MGI database lists 17 alleles of the mouse *Pax6* genes including homozygous viable and fertile hypomorphic alleles. The missense and nonsense mutations are summarized in Fig. 1b; it is surprising that most of the missense mutations are hypomorphs (five out of six), whereas among nine nonsense mutations just one is hypomorphic. Moreover, several splice-site mutations and minor or major deletions are listed in the MGI database. It might be of interest in our context here, that five *Pax6* alleles are associated with coloboma (*Del(2)Pax6*^*11Neu*^, *Pax6*^*132* − *14Neu*^, *Pax6*^*Sey* − *Dey*^, *Pax6*^*Sey* − *H*^, *Pax6*^*Sey*^ and *Pax6*^*tm1.2Xzh*^; http://www.informatics.jax.org/searchtool/Search.do?query=coloboma&page=featureList). This allelic series of different phenotypes indicates the need to investigate more than just a knockout of a given gene to see the full spectrum, of which mutations in a given gene might be responsible for—to model also the variability in human genetics.

*Pax6* is the vertebrate homolog of the eyeless gene (*ey*) in *Drosophila*. Since the mouse *Pax6* gene is able to ectopically express a compound eye at the antennae of *Drosophila*, the dogma of different routes in evolution for a vertebrate lensed eye vs an ommitidial eye in *Drosophila* was skipped at least for the underlying genetic regulation (Halder et al. 1995).

Moreover, in mammals, *Pax6* is expressed also in other organs, particularly in the brain and in the pancreas. In the brain, it is involved in the regulation of neurogenesis, cell proliferation and patterning effects (Haubst et al. 2004), and in the pancreas, it participates in the regulation of α-cell development (Dames et al. 2010). Therefore, *Pax6* might be considered also as a major example of pleiotropic effects. Similarly, *Bmp4* mutant mice have been considered as models for urinary system disease and atrioventricular septal defects, and *Bmp7* mutants as models for osteoarthritis (MGI database). However, in much more mutant lines affecting the genes discussed in this section, altered phenotypes are observed for many tissues and organs besides the eye.

## Microphthalmia

Microphthalmia or small eyes seem to be a more frequent, but also a rather variable phenotype in the mouse. The mammalian phenotype ontology annotations in the Mouse Genome Informatics (MGI) database contain 505 genotypes of 269 genes; 64 of them are unknown. There are significant overlaps with anophthalmia (e.g. mutation in *Pax6, Bmp4, Bmp7*) as well as with cataracts (e.g. mutations affecting genes coding for crystallins or connexins).

One of the early mouse mutants suffering from microphthalmia (gene symbol *mi*) was found by Paula Hertwig (1942) in a mouse cohort originating from irradiated mice; this particular mutation, however, most likely occurred spontaneously in the cohort (Arnheiter 2010). Today, we know that this mutation affects the ***Mitf*** gene coding for the **microphthalmia-associated transcription factor** (also referred to as melanogenesis-associated transcription factor). The *mi* mutation is characterized by the loss of an Arg residue at the C-terminal part of the DNA-binding domain (Hodgkinson et al. 1993). In the meantime, many alleles of *Mitf* have been described in the mouse, and the phenotypic range of the mutations is very broad—from dominant phenotypes as in the *Mi* allele to recessive phenotypes with almost no pathological effect (as in the spotted allele). Among them, three alleles are reported to be associated with colobomata. However, the microphthalmia phenotype occurs mainly in the homozygous mutants; since this transcription factor is expressed in many tissues and cell types including melanocytes, it affects also the skin or hair color. An excellent overview about this allelic series of the different mutations in the *Mitf* gene was published by Steingrímsson et al. (2004); it is a powerful example of the broad variability of disease-causing variations in a given gene.

**Fig. 2:**
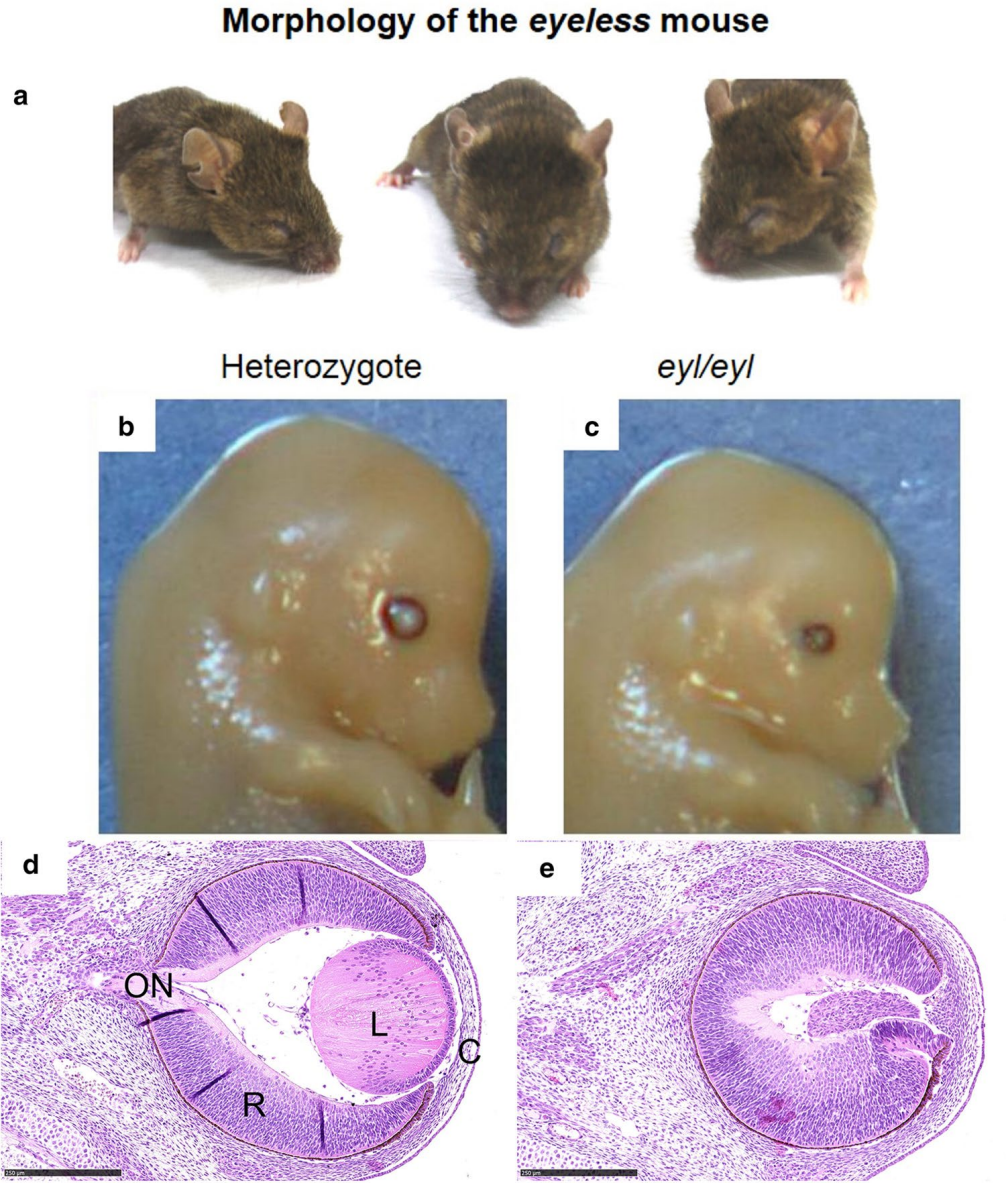
Morphology of the *eyeless* mouse—a model for microphthalmia. **a** The mutant mice have closed eyelids with very small eyes (microphthalmia). **b**–**e** Heterozygous and homozygous *eyeless* mutants are compared at embryonic day 14.5. In the upper panel, it is obvious that the eye of the homozygous mutant (**c**) is much smaller than the wild-type eye (**b**). In the lower panel, a transverse section through the eye is given (H/E staining): in contrast to the regularly formed wild-type eye (**d**), the eye of the homozygous mutants (**e**) is highly disorganized: the cornea is thicker, the lens is largely missing (only a remnant is present), and the retina is hypertrophic filling most of the vitreous body. Bar = 250 µm. *C* cornea, *L* lens, *ON* optic nerve, *R* retina. Modified, from Rosemann et al. 2010, with permission from Springer Science + Business Media, LLC 2009

Mutations in another gene coding for a transcription factor lead also to interesting phenotypic features—not only for microphthalmia. It is ***Pitx3***, coding for the **paired-like homeodomain transcription factor 3** (Fig. 2). The first mutant line, in which this gene was affected, was reported as aphakia (gene symbol *ak*); it is a spontaneous recessive mutation showing a severe microphthalmia in homozygous mutants (Varnum and Stevens 1968). Later, it turned out that the phenotype was caused by two major deletions in the promotor region of the *Pitx3* gene leading eventually to a classical null allele (Semina et al. 2000; Rieger et al. 2001). This mutation stops lens development at the stage of the lens vesicle; it does not detach from the surface ectoderm (the future cornea) and is degraded rapidly. The empty space is filled by hyperproliferating retinal tissue (Semina et al. 2000). The rapid degradation of the developing lens is due to reduced proliferation and increased apoptosis in the *ak* lenses (Medina-Martinez et al. 2009); Another allele (gene symbol: *eyl*—eyeless) of the mouse *Pitx3* gene is an insertion of a G after cDNA position 416 (416insG; exon 4). The shifted open reading frame is predicted to result in a hybrid protein still containing the PITX3 homeobox, but followed by 121 new amino acids. Since *Pitx3* is also expressed in the *substantia nigra* of the brain, this mutation affects also the formation of dopaminergic neurons in the *substantia nigra; Pitx3* mouse mutant lines are, therefore, an excellent model for Parkinson’s disease (Rosemann et al. 2010). Another spontaneous *Pitx3* mutant line (gene symbol *miak*—microphthalmia and aphakia) has a nonsense mutation (Tyr148X) leading to a truncation of PITX3 lacking the OAR domain (Wada et al. 2014); its phenotype is quite similar to *ak* and *eyl*.

Moreover, it turned out in later experiments that one of the target genes being regulated by PITX3 is *Foxe3*. The phenotype of a spontaneous *Foxe3* mutation, dysgenic lens (gene symbol *dyl*), resembles very much the *ak* phenotype, which can be easily explained by this interaction (Ahmad et al. 2013). Besides their phenotypical similarity, one of the interesting common features of the *Pitx3* and the *Foxe3* mutations is the loss of crystallin expression and the downregulation of *Prox1* (Anand and Lachke 2017). Since it is well known that PROX1 activates directly the expression of γ-crystallin genes (and SIX3 inhibits their expression; Lengler et al. 2001), we could consider an activating cascade from PITX3 to *Foxe3*; in turn, FOXE3 could activate *Prox1*, and when expressed, its encoded transcription factor leads to an increased γ-crystallin expression. It is a testable hypothesis explaining a “no lens phenotype”, and it would be interesting to see the entire network of this transcription factor-mediated signaling cascade. Since the mouse phenotype of *Pitx3* mutations is quite different from the human situation, in which dominant anterior-segment dysgenesis and cataracts are the predominant phenotypes of *PITX3* mutations, differences in this signaling cascade might be expected between mouse and man to explain this discrepancy.

In humans, the retinoic acid synthesis pathway including the genes *ALDH1A3* (aldehyde dehydrogenase 1 family, member A3; OMIM 600463), *RARB* (retinoic acid-binding receptor B; OMIM 601972) and STRA6 (stimulated by retinoic acid 6; OMIM 610745) was discussed being one of the major causes of microphthalmia or even anophthalmia (Williamson and FitzPatrick 2014). Surprisingly, in the mouse, mutations in these genes are not involved into corresponding clinical features. Several mouse mutant lines are available for *Aldh1a3*, and they suffer from various eye diseases, but microphthalmia is not a term, which is used to characterize their phenotype. In contrast, increased corneal stroma thickness or increased total retina thickness are some of their features (http://www.informatics.jax.org/marker/key/47335). Similarly, the mouse *Rarb* mutants show ocular disorders including colobomata and cataracts, but no microphthalmia or anophthalmia. Moreover, *Stra6* mouse mutants revealed malformations in the choroid and retinal pigmented epithelium; the length of the rod outer segments was shortened, and the number of cone photoreceptors was reduced. Interestingly, treatment of *Stra6*^−/−^ mice with pharmacological doses of vitamin A rescued their vision (Amengual et al. 2014). Taking these findings together, it suggests that there are major differences between mice and humans concerning the importance of the retinoic acid pathway for the eye development.

There is a further group of mouse mutants, which are characterized by similar types of **blebs** in combination with microphthalmia: blebbed (*bl*; Phillips 1970), head blebs (*heb*; Varnum and Vox 1981), eye blebs (*eb;* Chapman and Hummel 1963), myelencephalic blebs (*my*; Little and Bagg 1923), and fetal hematoma (*fh;* Center 1965); unfortunately, the *fh* mutant line became extinct before it could be molecularly characterized. The other four mutant lines carry mutations in genes coding for extracellular matrix proteins. The first one, *bl*, was identified in an F3 screen of a radiation genetics experiment and characterized by reduced eyes and clubbed feet. Later on, the reduced eyes were characterized as cryptophthalmos (fusion of eyelids), associated with distal limb defects. Finally, it turned out that the underlying mutation affects the *Fras1* gene (Fraser extracellular matrix complex subunit 1). The underlying mutation in the *bl*/*bl* mice was characterized as a nonsense mutation (7313C→A; S2200X) within the 47th exon of the *Fras1* gene (McGregor et al. 2003). The *Fras1* gene is expressed in many organs; its expression in the lens seems to be causative for the eye malformation in the *bl* mutant line. The *my* mutant line arose also in a radiation genetics experiment (Little and Bagg 1923) and exhibits a similar phenotype like *bl* (cryptophthalmos, and distal limb defects). By a series of genetic testing, this mutation was eventually mapped to *Frem2* (Fras1-related extracellular matrix protein 2), however, without showing the causative mutation in this particular gene (Jadeja et al. 2005). The *eb* mutants show also a recessive mode of inheritance and a similar phenotype like the *my* mutants (Chapman and Hummel 1963). Takamiya et al. (2004) demonstrated that a deletion of exons 10 and 11 of the *Grip1* gene (glutamate receptor interacting protein 1) is responsible for the eye bleb phenotype. The 4th mutant line of this series was *heb*, which is characterized by absent or malformed eyes; cryptophthalmos is always present in these mutants. The mutation in this particular line affects the gene *Frem1* encoding the Fras1-related extracellular matrix protein 1. The causative mutation is a LINE1 insertion 41 bp from the end of exon 17 (Smyth et al. 2004). In an ENU-induced mutation of similar phenotype (*bat*), the authors identified another causative mutation of the *Frem1* gene, close to the splice donor site of intron 25 leading to skipping of exon 25, a frame shift and a premature stop codon in exon 26. These mutant lines demonstrate the genetic heterogeneity of this phenotype, but they are now also considered being excellent models for the human Fraser syndrome, which is characterized as “cryptophthalmos with syndactyly” (Ramsing et al. 1990).

## Cataract

Cataract mutants in the mouse are easy to detect, either by the naked eye, if the lens opacity is total and severe, or by a slit lamp, which is a routine, non-invasive device in ophthalmology and since decades also applied in mouse ophthalmogenetics (Kratochvilova 1981). Therefore, the mammalian phenotype ontology annotations count a similar high number of cataract genotypes like for microphthalmia, namely 459 genotypes. Among them, there are 138 genes listed with targeted mutations, 54 genes with point mutations or small InDels (partially overlapping) and 50 loci with unknown mutations.

Before the onset of major genetic screens for eye diseases, there were some spontaneous cataract mutants reported. Dominant cataracts were the Fraser Cataract (*Cat*^*Fr*^; Fraser and Schabtach 1962), eye lens obsolescence (*Elo*; Oda et al. 1980), lens opacity (*Lop*; Lyon et al. 1981) or the Philly cataract (*Phil*, Kador et al. 1980). Among the recessive cataracts, the Nakano cataract (*nct*; Fukui et al. 1976) and the vacuolated lens (*vl*; Dickie 1967) were well known. In this group, the Philly cataract was the first, which was characterized at a molecular level: the mutation is a 12-bp deletion at the beginning of the 6th exon of the *Crybb2* gene (coding for βB2-crystallin; Chambers and Russell 1991).

The **crystallins** are highly concentrated and densely packed structural proteins in the eye lens; they are necessary for lens transparency. In mammals, we know two major crystallin families: the α-crystallin/small heat-shock protein family (consisting of two genes, *Cryaa* and *Cryab*), and the β/γ-crystallin “super”family (consisting of eight *Cryg* genes and six *Cryb* genes). Mutations in each of these genes lead to several forms of cataract; however, there is no genotype–phenotype correlation possible beside the association between the onset of the crystallin expression in the lens and the age of onset of the disease: cataracts caused by mutations in *Cryg* genes are visible at weaning (for a review see Graw 2009), but mutations in the *Crybb2* gene lead to a progressive cataract starting a few weeks after birth (Kador et al. 1980; Ganguly et al. 2008). Mutations in the *Cryb*/*Cryg* genes, usually, lead to a dominant phenotype; however, *Cryaa* mutations may cause dominant (*Aey7*: c371T→A, Val124Glu; Graw et al. 2001) or recessive cataracts (*lop18*: c161G→A; Arg54His; Chang et al. 1999).

In the past, the different crystallin-encoding genes were believed to be expressed in the lens only; however, we learned during the last years that many of these genes have also other functions outside the lens. The mouse mutations in the *Cryab* gene (encoding αB-crystallin) showed not only cataracts, but also other diseases, affecting mainly the heart, but also skeletal muscle fibers (Andley et al. 2011). Similarly, the gene of the major β-crystallin, *Crybb2*, is expressed in the testes leading to subfertility if mutated (DuPrey et al. 2007). *Crybb2* is also expressed in the brain (Magabo et al. 2000; Ganguly et al. 2008), and in homozygous *Crybb2* mutants, parvalbumin-positive interneurons as well as dendrites and dendritic branches in the hippocampus are decreased (Sun et al. 2013, 2018), and eventually changes in schizophrenia-related endophenotypes have been observed (Heermann et al. 2018).

Another major group of cataract mutations in the mouse affects the *Gja8* gene (gap junction α8) coding for **connexin50**, which is one of the components of lens gap junctions (besides connexin43 and connexin46; for review see Gong et al. 2007; Berthoud and Ngezahayo 2017). Point mutations in the *Gja8* gene lead to early-onset, dominant cataracts, whereas the knockout of this gene has a milder phenotype; however, since also gap junctions are formed by different combinations of the three connexins at different regions of the lens, the types of cataracts formed are highly diverse (Gong et al. 2007).

Among the spontaneously arisen cataract mutants, the *Cat*^*Fr*^ and *Lop* mutations have been shown to be allelic soon after their discovery. The molecular analyses revealed point mutations in the gene encoding the **major intrinsic protein** (*Mip*) of the lens (also known as aquaporin 0). The *Cat*^*Fr*^ mutation was demonstrated to be the result of a transposon-induced splicing error that substitutes a long terminal repeat sequence for the carboxy-terminus of MIP. The *Lop* mutation is characterized as a c151G → C exchange leading to a non-conservative exchange, Ala51Pro (Shiels and Bassnett 1996).

Besides structural proteins such as crystallins or membrane proteins, oxidative stress and perturbation in lens glutathione homeostasis are frequently discussed being causative mechanisms for cataract formation (for reviews see Lou 2003; Fan et al. 2017). Along this line, a mutation (c3816T→A) in the *Pxdn* gene encoding peroxidasin was characterized leading to a premature stop codon. Morphologically, the mutant mice suffer from congenital cataracts because of a disorganized lens matrix and ruptures of the lens capsule; lens cells are present in the anterior chamber as well as in the posterior vitreous body (Yan et al. 2014).

The **recessive cataract mutations**, *nct* and *vl*, have been characterized moleculary, too: The Nakano cataract (*nct*) is characterized by a mutation affecting the *Cpox* gene encoding the coproporphyrinogen oxidase (Mori et al. 2013). The mutation *vl* was eventually characterized by an 8-bp deletion affecting the gene *Gpr161* coding for a G-coupled receptor (Matteson et al. 2008).

**Fig. 3:**
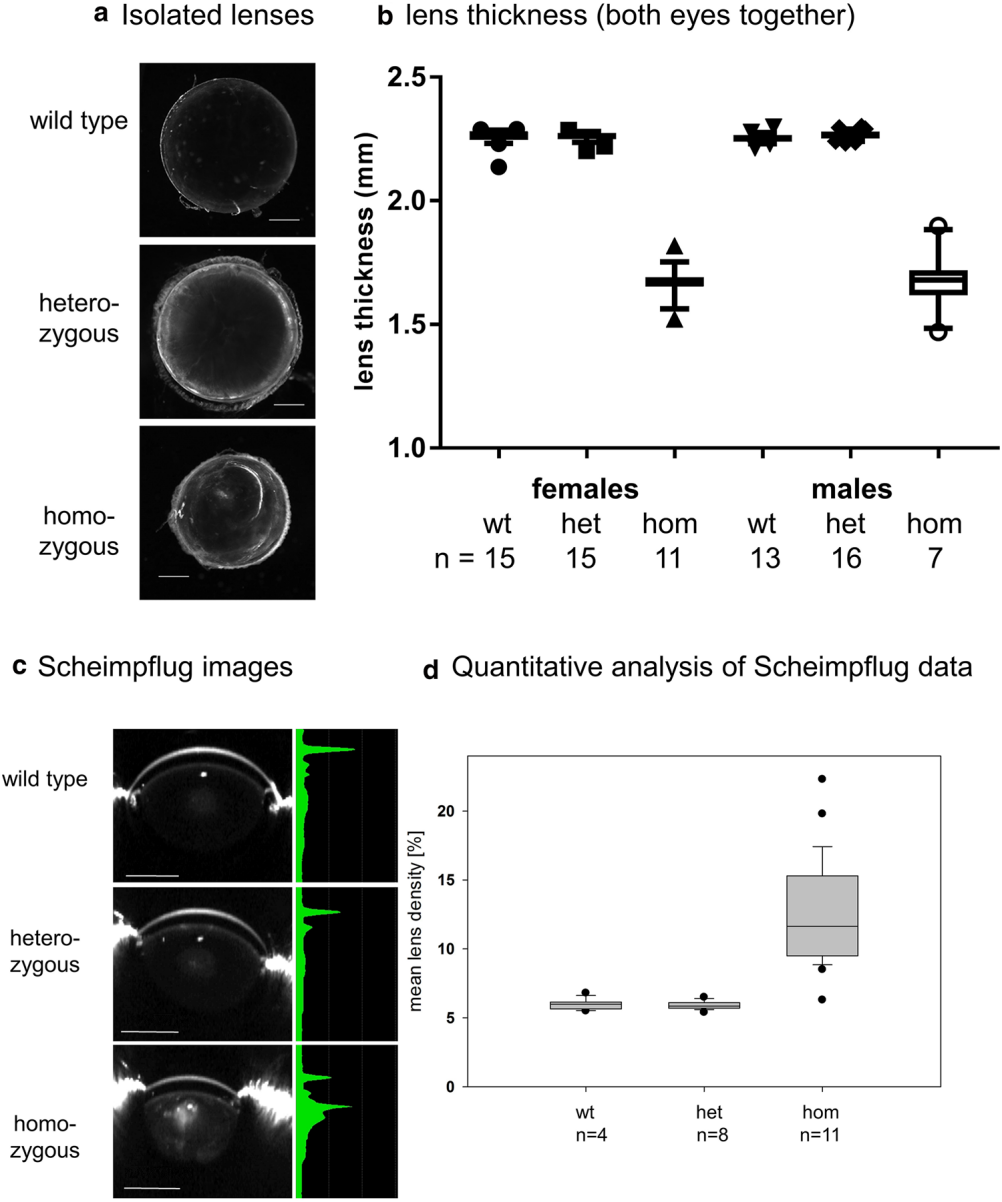
Cataracts in *Ercc2*^*Rco015*^ mutant mice. **a** Lenses of 5-week-old wild types, hetero- and homozygous *RCO015* mutants are prepared and photographed. The lenses of wild types are completely clear; the lenses of heterozygous mutants demonstrate opacities at the capsule, and in the homozygous mutants clear boundaries in the cortical areas are observed in addition to the nuclear opacity. The lenses of homozygous mutants are smaller (bar: 500 µm). **b** The lens thickness was determined using the established optical low coherence interferometry technique (Puk et al. 2006). It turned out that the lenses of the homozygous *Ercc2* mutants (both males and females at the age of 25 weeks) are more than 20% smaller (*p* < 0.001) than the lenses of heterozygous mutants or wild-type littermates. The quantitative data of the lens thickness are given in a box-and-whisker plot; the whiskers give the 1st and 3rd quartiles, and the bar in the middle of the box indicates the median of the axial length. (Oana Amarie, unpublished data of the German Mouse Clinic, GMC). **c** Scheimpflug imaging of the same lenses as shown in **a** demonstrates the clear lenses in wild types and heterozygotes; the opacity in the nuclear region of homozygous mutants is clearly visible above the slightly opaque background of the entire lens (bar: 1000 µm). **d** The quantitative data of the lens density of the Scheimpflug images are given in a box-and-whisker plot; the whiskers give the 1st and 3rd quartiles, and the bar in the middle of the box indicates the median of the lens density (from Kunze et al. 2015, with permission from the authors)

Cataractous lenses of the mouse are frequently reported to be smaller than wild-type lenses (e.g. Graw et al. 1990a, b, 2004). Since the lens defines the size of the entire eye, also the entire eye is obviously smaller. Correspondingly, there is a group of mouse mutants, which are characterized as heterozygotes by a smaller size of the lens as determined by laser-interference biometry (Puk et al. 2006). At least some of these mutants develop cataracts later in life (e.g. mutation in *Cryba2*, encoding βA2-crystallin; Puk et al. 2011a) or in homozygotes [(e.g. mutation in *Lim2*, encoding the lens intrinsic membrane protein 2; Puk et al. 2011b, or a mutation in *Ercc2* (excision repair cross-complementing rodent repair deficiency, complementation group 2); Kunze et al. 2015; Fig. 3]. Therefore, it would be an interesting question, whether a reduced lens size might be an early biomarker for cataract formation at later stages in life. Mouse mutants are a valuable tool to test this hypothesis.

In addition to mouse models for congenital, childhood or juvenile cataracts, a few mutant lines exist also for senile cataracts. One group of such mutants is the senescent-accelerated mice (SAM) having been developed at the Kyoto university since 1970 (Takeda et al. 1981). Two of the SAM-lines develop also cataracts (SAM1P, Nishimoto et al. 1993; SAM9P; Ashida et al. 1994). Unfortunately, the underlying mutation(s) have not yet been described for these mutant lines. Similarly, the EMORY mouse is also well recognized as a genetic model for age-related cataracts (Kuck et al. 1981–1982). There were several interesting ultrastructural and biochemical data reported like the specific upregulation of the adhesion-related kinase (AKR; Sheets et al. 2002), or—more recently—regional changes of AQP0-dependent square array junction and gap junctions (Biswas et al. 2014), but also for this mutant line the underlying mutation for the dominant mode of inheritance (Kuck et al. 1981/1982) remains to be elaborated.

## Missing mutants and unknown mutations

Analyzing the Mouse Genome Informatics (MGI) database on mouse mutants (http://www.informatics.jax.org/) for genes involved in anophthalmia, microphthalmia and cataracts, it is obvious that a remarkable number of discovered and phenotypically described mutants is not yet characterized at the molecular level (Table 1). Unfortunately, some of the older mouse lines are already extinct, but others are still available. Using the advanced sequencing techniques including whole exome sequencing, resolving of the remaining “old” mutant lines should be possible fast.

**Table 1:** Unsolved mutants for anophthalmia/microphthalmia and microphthalmia/cataract

Gene symbol	Anophthalmia/microphthalmia	Microphthalmia/cataract	Strain available (if and where)
*Alm*		Anterior lenticonus with microphthalmia	Extinct
*B2b1511*	Anophthalmia, microphthalmia		JAX
*B2b1702*	Anophthalmia, microphthalmia		Extinct
*B2b1963*	Anophthalmia, microphthalmia		JAX
*B2b2012*	Cyclops, anophthalmia, microphthalmia		Extinct
*B2b2110*	Anophthalmia, microphthalmia		JAX
*B2b2153*	Enophthalmia, anophthalmia, microphthalmia		JAX
*B2b2739*	Anophthalmia, microphthalmia		JAX
*bh*	Brain hernia	Brain hernia	Extinct
*Bld*		Blind	Extinct
*Cat3*		Cataract 3	HMGU
*dblr*	Doubleridge		Extinct
*dcm*		Dense cataract and microphthalmia	Extinct
*eob*		Eye lids open at birth	Extinct
*exma*	Exencephaly with severe microphthalmia/anophthalmia		Extinct
*ey2*	Eyeless 2		Extinct
*ey3*	Eyeless 3		JAX
*ey4*	Eyeless 4		JAX
*eyl2*	Eyeless 2 Jackson		JAX
*Iac*		Iris anomaly with cataract	Extinct
*Idc*		Iris dysplasia with cataract	Extinct
*jrc*		Juvenile recessive cataract	Extinct
*Lcl*		Lens cloudy	Harwell, EMMA
*lg*	Lid gap	Lid gap	Extinct
*nmf131*		Cataract, microphthalmia	JAX
*Pcs*		Polar cataract and small eyes	Extinct
*Rgsc258*		Cataract, microphthalmia	RIKEN
*Rgsc152*		Cataract, microphthalmia	RIKEN
*Rgsc1371*		Cataract, microphthalmia	RIKEN
*Rgsc1465*		Cataract, microphthalmia	RIKEN
*Tcm*		Total cataract with microphthalmia	Extinct
*tirs*	Tiresias		Extinct

Data are from the Mammalian Phenotype Ontology Associations of the Mouse Genome Informatics database (http://www.informatics.jax.org/vocab/mp_ontology) using the search terms “anophthalmia”, “microphthalmia” and “cataract” (Sept. 25, 2018)

Resources for mouse mutants:

*EMMA* The European Mouse Mutant Archive; c/o Helmholtz Center Munich, Institute of Experimental Genetics; Neuherberg/Germany; https://www.infrafrontier.eu/search

*HMGU* Helmholtz Center Munich, Institute of Developmental Genetics; Neuherberg/Germany; http://www.helmholtz-muenchen.de/en/idg/research/neuropsychiatric-diseases/eye-disease/research/index.html

*JAX* The Jackson Laboratory, Bar Harbor, USA; https://www.jax.org/orderform

*RIKEN* Riken BioResource Research Center, Tsukuba/Japan; http://mus.brc.riken.jp/en/order

*Harwell* MRC Harwell; Harwell Science and Innovation Campus, Harwell/UK; http://www.mousebook.org/stock-list

Nevertheless, comparing the numbers of genes identified in the mouse for eye diseases discussed here with the genes affected in humans, the number of mouse genes is much lower. For cataracts, the CatMap (https://cat-map.wustl.edu/) lists 324 cataract genes in humans, but the corresponding list of the mouse comprises just the half or 50% of the human cataract genes still need a mouse model. Unfortunately, no similar databases exist for genes involved in human anophthalmia or microphthalmia. The modern CRISPR/Cas9 technology (Knowlton and Smith 2017) offers one efficient possibility for designing new mutant alleles for interesting disease-causing mutations. Another option would be searching an archive of mouse mutations induced by ENU (e.g. https://www.helmholtz-muenchen.de/ieg/services/scientific-resources/index.html), re-deriving the mutants from frozen sperms and checking for the suggested phenotype.

Mouse mutant lines are today also systematically analyzed for their phenotypes (http://www.mousephenotype.org/). Worldwide, 18 institutions are collaborating in the International Mouse Phenotyping consortium (http://www.mousephenotype.org/about-impc/impc-members) screening also for eye anomalies. First results have been published recently (Moore et al. 2018), and actual results can be found on the IMPC website looking for “eye morphology”. However, there is no way to check directly for terms such as anophthalmia, microphthalmia or cataract. Instead, mouse mutations can be found on the MGI database searching for diseases discussed in this review using the corresponding gene-ontology terms (http://www.informatics.jax.org/vocab/gene_ontology).

Finally, the mouse offers the possibility to genetically test also for the roles of modifiers and for the role of SNPs—these aspects open new avenues for ophthalmogenetics in the mouse.

## Conclusions

A wide range of mouse models for microphthalmia, anophthalmia and cataracts have been described in detail and molecularly characterized. However, for many mouse mutant lines the underlying mutation still needs to be identified. However, for many genes, which have been shown to be involved in human anophthalmia, microphthalmia or cataracts, the corresponding mouse model still needs to be established.
